# A word of thanks to our reviewers in 2017 and a call for new reviewers

**DOI:** 10.1080/13814788.2018.1454766

**Published:** 2018-04-26

**Authors:** Jelle Stoffers, Carl Llor, Manfred Maier, An de Sutter, José Maria Valderas, Henk van Weert, Adam Windak

**Affiliations:** Editors, The European Journal of General Practice

The Editors of the *European Journal of General Practice* (EJGP) would like to express their gratitude to all reviewers who have advised us during the year 2017. To acknowledge their essential contribution to the EJGP, we have listed the names of all referees who completed at least one review report in 2017.

Abholz, Heinz-Harald; Agyapong, Vincent IO; Ak, Filiz; Alastrué Loscos, Ignacio; Antonneau, Camilla; Apolinario, Daniel; Arestedt, Liselott; Ariman, Osman; Ataoglu, Esra; Avellaneda Fernández, Alfredo; Avignon, A; Bailey, Richard; Baker, Richard; Bally, Klaus; Banegas, Jose R; Barreto, Philipe; Barton, Christopher; Bedford, Helen; Benko, Ria; Bennasar, Miquel; Berendsen, Anette; Berkhout, Christophe; Berking, Matthias; Bindels, Patrick; Birner, Peter; Black, Laurie; Bleeker, Joan; Bobe-Armant, Francesc; Both, Stephanie; Bouma, Margriet; Boussageon, Rémy; Bowie, L; Branch, Andrea D; Brismar, Kerstin; Brodbeck, Jeannette; Brotonos, Carlos; Bucher, Sophie; Buczkowski, Krzysztof; Buil, Pilar;Buntinx, Frank; Buring, Julie; Cals, Jochen; Cameron, Isobel; Campbell, Eric; Campiñez, Manuel; Cardy, Amanda; Carter, Ben; Castro, Yessenia; Čatić, Tarik; Cavadas, Luís; Celebi, Nora; Chatelard, Sophia; Chmiel, Corinne; Chovarda, Eleni; Clare, Bradley; Clarke, Amy; Cobb, Kate A; Cobley, Stephen; Coindard, Guillaume; Coll, Josep; Collington, Val; Cox, Anna; Czachowski, Slawomir; Dahlhaus, Ann; Daker-Smith, Graham; Damoiseaux, Roger; Damsted Petersen, Christina; Davidsen, Annette Sofie; de Jager, Eloise; De Kock, Cornelis; De la Figuera von Wichmann, Mariano; De Ly, Yasmien; de Sutter, An; de Vries, Juriena; De Wet, C; Degryse, Jan; Del-Pino-Casado, Rafael; Diaz, Esperanza; Donker, Ge; Dowrick, Chris; Driot, Damien; Drosdzol-Cop, Agnieszka; Dunne, Suzanne; Dwinger Sarah; Ebell, Mark H; Edwards, Adrian GK; Eizenga, Wietze; Elliot, Elizabeth; Enrique Dominguez-Munoz, J; Esmer, Aytul Corbacioglu; Falcato, Luis; Farrell, Elinor H; Farris, Samantha G; Fernandez, Rosemarie; Fischer, Gabriele; Fleury, Marie-Josee; Flin, Rhona; Flocke, Susan; Fortin, Martin; Fransen, Mirjam P; Gaakeer, Menno; Gabrielli, Maurizio; Gagyor, Ildiko; Gaist, David; Galvez, Luis; Gappmaier, Eduard; García-Pérez, Sonia;Garvey, Chris; Geelen, Els; Gehring, Katrin; George, Michael; Gervas, Juan; Gibson, Peter; Gidman, Wendy Gillian S, Stephens; Girdley, Forrest; Gjeraa, K; Glynn, Liam; González-Juanatey, José; Gould, Dinah; Gouveia, Alexandre; Grande, David; Greenhalgh, Trisha; Gryglas, Anna; Gucek, Nena; Guerra Pérez, María Teresa; Güldal, Dilek; Gunal, Ozgur; Gustafsson, Lars L; Guthrie, Bruce; Haastrup, Peter; Halcomb, Elizabeth; Hallinger, Philip; Harmsen, Charlotte Gry; Harvey, Jasmine; Haukoos, Jason S; Hayes, Risa P; Heaslip, Vanessa; Heddini, Ulrika; Herzig, Lilli; Hetlevik, Øystein; Heyland, Daren; Hoffmann, Kathryn; Holmgren, Kristina; Holzinger, Anita; Iljaz, Rade; Inoue, Machiko; Ippolito, Ernesto; Jaatinen, Pekka; Jacobi, Arnd; Jacobs, Lenworth M; Jaffray, Mariesha; Jamouille, Marc Jayasuriya, Rohan; Johnson, Crista; Jones, Roger, Junius-Walker, Ulrike; Jurado, Dolores; Kaae, Susanne; Kahan, Thomas; Kalabay, Laszlo; Kalda, Ruth; Kall, Meaghan; Kamenski, Gustav; Kane, Bridget; Kangovi, Shreya; Karnilowicz, Wally; Keizer, Ellen; Kelly, Brian; Kennedy, Suzanne; Khairy, Amal; Kim, Hung Jeffrey; Kinmonth, Ann Louise; Klemenc-Ketis, Zalika; Klemp, Kerstin; Klimas, Jan; Knight, Andrew; Kontopantelis, Evan; Korhonen, Päivi; Korstjens, Irene; Kouladjian O'Donnell, Lisa; Kraaijvanger, Nicole; Kreber, Carolin; Kuang-Ho, Chen; Kutalek, Ruth; Laner, Birgit; Laurence, Caroline; Le Reste, Jean-Yves; Leeflang, Mariska; Liddy, Clare; Lischka, Martin; Lopez-Picazo, Julio; Lowres, Nicole; Lowrie, Richard; Lowthian, JA; Luc, Deliens; Lucassen, Peter; Lunze, Karsten; Maagaard, Roar; Maddern, Guy; Magin, Parker; Mäkelä, Marjukka; Mallen, Christian; Marakoğlu, Kamile; Marcinowicz, Ludmila Marinho, José; Marzo-Castillejo, Mercè Mas, E; Mascort Roca, Juanjo; Mathieu, Sylvain; McAllister Marion; McNaughton, Candace D; Meagher, David; Medina Bombardo, David; Miedema, Baukje; Milos, Veronica; Mol, Saskia; Mola, Ernesto; Monshouer, Karin; Moragas-Moreno, Ana; Motl, Robert W; Mounce, Luke; Munck, Anders; Muraro, Antonella; Murchie, Peter; Murtagh, Jessica; Muth, Christiane; Naimer, Sody; Narendran, Parth; Navickas, Rokas; Nelson, Brett; Newham, Rosemary; Nicholas Steel; Nielen, Markus; Norgaard, Birgitte; Nygaard, Peter; Obimakinde, Abimbola; Ocek, Zeliha; O'Connor, Kieron; Ogose, Akira; Olsman, Erik; Olsson, Erika; Ornello, Rafaello; Ozyurek, Eser; Paget, John; Palka, Malgorzata; Patel, Asmita; Paul, Christine L; Pennington, Richard; Peremans, Lieve; Perl, Sabine; Petek Ster, Marija; Petrella, Robert; Pizzanelli, Miguel; Polomen, Viola; Pujol, Jesus; Pujol-Ribera, Enriqueta; Rasul, Saima; Razavi, Darius; Reed, Barbara; Regamey, Frederic; Reinisch, Walter; Reis, Shmuel; Reissing, Elke; Remmen, Roy; Renzi, Cristina; Ricci-Cabello, Ignacio; Richter, Trevor; Rifel, Janez; Rifel, Janez Janez; Rifkin, Alan; Rigal, Laurent; Rijpsma, Douwe; Roca Saumell, Carme; Rosemann, Thomas; Rosen, Natalie; Rosendal, Marianne; Ross, Jim; Rotaeche, Raf; Rotar Pavlič, Danica; Rouge-Bugat, Marie-Eve; Rowe, Brian H; Rowen, Tami; Rurik, Imre; Rutten, Guy; Saari, Henna; Sacco, Simona; Saint-Lary, Olivier; Sakayama, Kenshi; Saletti-Cuesta, Lorena; Samet, Jeffrey; Sandholzer, Hagen; Schellevis, Francois; Scherpbier, Nynke; Scherphof, Charlotte S; Schildmeijer, Kristina; Schmiemann, Guido; Schneider, Nils; Schnell, Oliver; Schoenmakers, Birgitte; Schomerus, Georg; Schrans, Diego; Seamark, David; Seltenhammer, Monika; Senn, Oliver; Senter, Carlin; Shojaa, Mahdieh; Silbernagl, Marisa; Simmons, Kecia; Simons, Sereh M. J; Siriwardena, Niro; Sisó Almirall, Antoni; Skogan, Örjan; Slight, Sarah; Smeele, Ivo; Smith, Susan; Smits, Marleen; Song, T. Ted; Sonnichsen, Andreas; Sørensen, Kristine; Spiegel, Wolfgang; Spoelstra, Symen K; Sunaert, Patricia; Svab, Igor; Szatkowski, Lisa; Szczesniak, D; Tejedor-Alonso, MA; Terluin, Berend; Thornton, Jane; Tod, Angela; Tomasik, Tomasz; Torloni, Maria Regina; Trappenburg, Jaap; Tvete, Ingunn; Twigg, Michael J; Uijen, Anne-Marie; Uittenbogaart, Steven; Vadiee, Massod; van Achterberg, Theo; van Bokhoven, Marloes; van Boxtel-Wilms, Susanna; van den Berg, Michael; Van Den Broucke, Stephan; Van Den Bruel, An; van den Eijnden, Regina; van der Heide, Iris; van der Horst, Henriëtte; Van der Weijden, Trudy; van der Wouden, Johannes; van Hunsel, Florence; Van Marwijk, Harm; Van Royen, Paul; van Schayck, Constant; Van Scoy, Lauren; van Weel- Baumgarten, Evelyn; Vannier, Sarah; Varnam, Robert; Verdú, Fernando; Vermandere, Mieke; Verstappen, Wim; Vicens, Caterina; Vikum, Eirik; Vinyoles, Ernest; Visser, Femke; Vogelsang, Harald; Vollebergh, Wilma; Voltmer, Edgar; Wallace, Emma; Wancata, Johannes; Wandeler, Gilles; Wändell, Per; Weinberger, Andrea H; Wensing, Michel; Wermeling, Paulien R; Whatley, Ian; Widhalm, Kurt; Widmer, Daniel; Williams, Pauline; Wojczewski, Silvia; Woo, Jean; Yengil, Erhan; Yuguero Torres, Oriol; Yun, Haesun; Zill, Jördis.

We want to thank you all for your assistance! Your review reports were valued feedback to the authors and helped us to make our editorial decisions.

Although our database of authors and reviewers steadily increases, the Editors do not always find it easy to find referees within the journal’s deadlines. Therefore, we sincerely hope that all reviewers want to continue their review work in the future. We provide a format to facilitate the review.

If you would like to update your reviewer account, e.g. to specify your expertise, please visit https://mc.manuscriptcentral.com/EJGP and log in to modify your profile. We also invite readers of the EJGP who would like to review manuscripts for this Journal, to visit http://mc.manuscriptcentral.com/ejgp and to register as ‘new user’, i.e. to complete the details of their expertise. Thank you!

If you have comments regarding the Journal or its peer review process, we invite you to contact the Editorial Office (Ms Anneke Germeraad-Uriot, Editorial Assistant, e-mail: ejgp-agermeraad@maastrichtuniversity.nl).

